# Gene Expression Deregulation in Postnatal Skeletal Muscle of TK2 Deficient Mice Reveals a Lower Pool of Proliferating Myogenic Progenitor Cells

**DOI:** 10.1371/journal.pone.0053698

**Published:** 2013-01-14

**Authors:** João A. Paredes, Xiaoshan Zhou, Stefan Höglund, Anna Karlsson

**Affiliations:** 1 Division of Clinical Microbiology, Department of Laboratory Medicine, Karolinska Institute, Karolinska University Hospital Huddinge, Stockholm, Sweden; 2 Department of Chemistry – BMC, Uppsala University, Uppsala, Sweden; New Jersey Medical School, University of Medicine and Dentistry of New Jersey, United States of America

## Abstract

Loss of thymidine kinase 2 (TK2) causes a heterogeneous myopathic form of mitochondrial DNA (mtDNA) depletion syndrome (MDS) in humans that predominantly affects skeletal muscle tissue. In mice, TK2 deficiency also affects several tissues in addition to skeletal muscle, including brain, heart, adipose tissue, kidneys and causes death about 3 weeks after birth. We analysed skeletal muscle and heart muscle tissues of *Tk2* knockout mice at postnatal development phase and observed that TK2 deficient pups grew slower and their skeletal muscles appeared significantly underdeveloped, whereas heart was close to normal in size. Both tissues showed mtDNA depletion and mitochondria with altered ultrastructure, as revealed by transmission electron microscopy. Gene expression microarray analysis showed a strong down-regulation of genes involved in cell cycle and cell proliferation in both tissues, suggesting a lower pool of undifferentiated proliferating cells. Analysis of isolated primary myoblasts from *Tk2* knockout mice showed slow proliferation, less ability to differentiate and signs of premature senescence, even in absence of mtDNA depletion. Our data demonstrate that TK2 deficiency disturbs myogenic progenitor cells function in postnatal skeletal muscle and we propose this as one of the causes of underdeveloped phenotype and myopathic characteristic of the TK2 deficient mice, in addition to the progressive mtDNA depletion, mitochondrial damage and respiratory chain deficiency in post-mitotic differentiated tissue.

## Introduction

Replication of nuclear DNA requires a large amount of deoxyribonucleoside triphosphates (dNTPs) and accordingly dNTP synthesis is strongly up-regulated in cycling cells. However, continuous mitochondrial DNA (mtDNA) synthesis in post-mitotic cells places a requirement of generating precursors for DNA synthesis also outside of S-phase. In eukaryotic cells, mtDNA represents 1–5% of total DNA in the cell and only a relatively small fraction of dNTPs is required for its replication. Early evidence suggested tight spatial separation of mitochondrial and cytosolic dNTP pools [1], [2], but more recent work has shown that mitochondrial and cytosolic dNTP pools are in fact rapidly mixed [3], [4].

dNTPs are generated via two pathways: the *de novo* pathway and the salvage pathway [5]. Cycling cells have a high rate of cytosolic *de novo* production catalyzed by ribonucleotide reductase (RNR). Also high activities of the cytosolic salvage pathway enzymes thymidine kinase 1 (TK1) and deoxycytidine kinase (dCK) are present in cycling cells. Post-mitotic cells have much lower levels of dNTP pools, and their synthesis rely heavily on the mitochondrial salvage pathway. In the salvage pathway, deoxyribonucleosides, derived from external sources or recycled within the cell, are phosphorylated and reused in DNA synthesis. The first phosphorylation step is rate limiting, and is performed by TK1 and dCK in cytosol and by thymidine kinase 2 (TK2) and deoxyguanosine kinase (DGUOK) in mitochondria [6]. TK1 phosphorylates deoxythymidine (dThd) and deoxyuridine (dUrd), and dCK phosphorylates deoxycytidine (dCyd), deoxyadenosine (dAdo) and deoxyguanosine (dGuo). Of the mitochondrial kinases, TK2 catalyzes phosphorylation of dThd, dCyd and dUrd while DGUOK phosphorylates dGuo and dAdo. Inactivating mutations in *TK2* or *DGUOK* severely compromise mtDNA maintenance and lead to mtDNA depletion syndromes (MDS) in humans [7]. *TK2* mutations primarily cause myopathic MDS [8]–[10], a form that presents in early infancy as feeding difficulty, failure to thrive, hypotonia and muscle weakness. Serum creatine kinase is often elevated, and the disease typically causes early death. *DGUOK* mutations are associated with a hepatocerebral form of MDS that typically presents with failure to thrive, vomiting, hypotonia and hypoglycemia [11].

MDS pathologies, and other kinds of mtDNA-associated diseases caused by primary DNA mutations or by defects in nuclear-encoded mtDNA maintenance proteins, usually lead to a deficient oxidative phosphorylation and to an insufficient ATP production, which does not explain why there are several types of different diseases instead of just one [7]. Virtually every organ system can be affected during mitochondrial disease, but tissues with high requirements for oxidative energy metabolism, such as muscle, heart, eye and brain are particularly vulnerable. Other cells, such as lymphocytic cells and skin cells seem to handle TK2 deficiency without major problems.

We have taken advantage of the thymidine kinase 2 knockout (*Tk2* ^−/−^) mouse model previously created in our laboratory [12] in order to further elucidate the cellular responses to lack of TK2. These TK2 deficient mice essentially develop normally for the first week but from then on exhibit a severe ataxic phenotype, growth retardation, pronounce hypothermia and die within 3 to 4 weeks of life. These mice also exhibit progressive mitochondrial DNA (mtDNA) depletion in several organs but without increased mtDNA mutations or structural alterations [12]. A similar picture was observed in the *Tk2* (H126N) knock-in mouse, which developed rapidly progressive weakness after age 10 days and died within 3 weeks. These mice showed unbalanced dNTP pools, mtDNA depletion and defects of respiratory chain enzymes containing mtDNA-encoded subunits that were most prominent in central nervous system [13]. Encephalomyopathy, neuronal dysfunction and generalized neurological impairment have also been observed in TK2 deficient mice [12]–[14], as well as an abnormal development and affected endocrine properties of adipose tissues [15]. It has been proposed that organ specificity and resistance to pathology in *Tk2* mutant organs depend on transcriptional compensation to the reduced mtDNA level, which seems to be the case in heart, but not in brain [16].

In the present study, skeletal muscle from the hind limb and heart muscle tissue from wild-type (*Tk2* ^+/+^) and *Tk2* ^−/−^ mice in postnatal development stage were compared in terms of growth, mtDNA levels, mitochondria phenotype and gene expression. We concluded that deficiency in the mitochondrial pyrimidine deoxyribonucleoside salvage enzyme led to mtDNA depletion, mitochondrial abnormalities and pronounced gene deregulation in both skeletal muscle and heart muscle tissues. The results also demonstrate that TK2 deficiency affects the function of myogenic progenitor cells in postnatal skeletal muscle tissue, which can be associated with slow muscle growth and slow development in *Tk2* knockout mice.

## Results

### TK2 deficient mice grow slower than the wild-type counterparts

Immediately after birth, *Tk2* knockout pups (*Tk2* ^−/−^) were not distinguishable from their wild-type (*Tk2* ^+/+^) or heterozygous (*Tk2* ^+/−^) litter counterparts. However after 14 days, *Tk2* ^−/−^ mice showed pronounced growth retardation. The body weight at this stage was 52% of the *Tk2* ^+/+^ mice's weight (Table 1). The average weight of the *Tk2* ^−/−^ mice analysed was 4.2±0.4 g, while for *Tk2* ^+/+^ mice the average weight was 8.2**±**0.4 g. The average weight of individual organs was always lower in *Tk2* ^−/−^ 14 days-old mice as compared to *Tk2* ^+/+^ mice, but for heart the decrease was not statistically significant (Table 1). Also, when we compared relative weights of different organs to total body weight, we observed that relative weight of *Tk2* ^−/−^ hearts was higher compared to skeletal muscle or liver, which was used as a non-muscular control tissue. In fact, when we compared the size of hearts and hind limbs (skeletal muscle) of 14 days-old *Tk2* ^+/+^ and *Tk2* ^−/−^ mice (Figure S1A), we observed that hearts were similar in size whereas the limbs of *Tk2* ^−/−^ mice were much smaller, thus it was clearly visible that heart tissue did not decrease in growth as skeletal muscle did. Although all muscular tissues are high energy demanding tissues, and consequently highly dependent on mitochondria metabolism, the observed differences between heart and skeletal muscle in response to the same enzyme deficiency led us to further analysis.

**Table 1:** Body and organs weights in 14 days-old *Tk2* ^+/+^ and *Tk2* ^−/−^ mice.

	*Tk2* ^+/+^	*Tk2* ^−/−^	% of *Tk2* ^+/+^
**Total body (g)**	**8.21±0,38**	**4.27±0,40** ***	**52.0%**
**Hind limb (skeletal muscles + associated bones) (g)**	405.10±25.19	231.67±35.50 **	57.2%
**Heart (g)**	45.80±4.61	39.03±4.20	85.2%
**Liver (g)**	309.53±14.26	164.83±18.79***	53.3%
**Hind limb/Total body**	**4.90±0.49**	**4.61±0.61**	
**Heart/Total body**	**0.55±0.04**	**0.78±0.10**	
**Liver/Total body**	**3.73±0,05**	**3.28±0.30**	

For each measurement, data from at least 3 different mice have been used (*n*≥3).The *P*-values for statistical comparisons (two-tailed unpaired Student's t-test) between *Tk2* ^+/+^ and *Tk2* ^−/−^ body or tissues weights are shown - **P<0.01;

*** *P*<0.001.

### Levels of mtDNA in skeletal muscle and heart during postnatal development

We compared mtDNA copy number per diploid genome in knockout (*Tk2* ^−/−^) muscle tissues with *Tk2* ^+/+^ tissues, during the same phase of postnatal development and observed that mtDNA depletion in *Tk2* ^−/−^ heart and skeletal muscle increased along the stage of postnatal development analysed (from 4 to 14 days after birth). Some mtDNA copy number decrease was also detected during this phase in *Tk2* ^+/+^ muscle tissues, but depletion was always more pronounced in *Tk2* ^−/−^ mice tissues. During the first 4 days of life, depletion relative to *Tk2* ^+/+^ was not detected, but the number of mtDNA copies was already different at the age of 8 days (55.0% of *Tk2* ^+/+^ mtDNA content in skeletal muscle and 60.3% in heart) and the difference was even bigger at the age of 14 days (26.8% in skeletal muscle and 30.6% in heart) (Table 2). Compared to day 4, where no statistically significant difference was detected, mtDNA copy number remaining in skeletal muscle and heart at day 14 decreased in both, *Tk2* ^+/+^ and *Tk2* ^−/−^ tissues, but that decrease was much higher for *Tk2* ^−/−^ tissues (Table 2). We also observed that after postnatal development (21 days), *Tk2* ^+/+^ skeletal muscle tissue had much lower levels of mtDNA copies as compared to *Tk2* ^+/+^ heart tissue. At 2 months of age, heart contained about 25000 mtDNA copies per diploid nucleus, about 5 times more than the approximately 5000 copies per nucleus present in skeletal muscle (data not shown).

**Table 2:** mtDNA copy number per diploid nucleus in skeletal muscle and heart of *Tk2* ^+/+^ and *Tk2* ^−/−^ pups at 4, 8 and 14 days after birth.

	day 4	day 8	day 14	% of mtDNA (day 14 *vs.* day 4)
Skeletal muscle *Tk2* ^+/+^	2597±517	2766±306	1289±694	**49.6%**
Skeletal muscle *Tk2* ^−/−^	2491±151	1522±485**	345±81 *	**13.8%**
**% of mtDNA (** ***vs. Tk2*** **^+/+^)**	**95.9%**	**55.0%**	**26.8%**	**-**
Heart *Tk2* ^+/+^	4431±521	4491±1150	3131±1029	**70.7%**
Heart *Tk2* ^−/−^	3431±1316	2706±803 *	958±842 *	**27.9%**
**% of mtDNA (** ***vs. Tk2*** **^+/+^)**	**77.4%**	**60.3%**	**30.6%**	-

For each time point, data from at least 3 different mice have been used (*n*≥3). The *P*-values for statistical comparisons (two-tailed unpaired Student's t-test) between *Tk2* ^+/+^ and *Tk2* ^−/−^ tissues are shown - *P<0.05;

** *P*<0.01.

### Mitochondria ultrastructure is altered in TK2 deficient mouse muscle tissues

**Figure 1:**
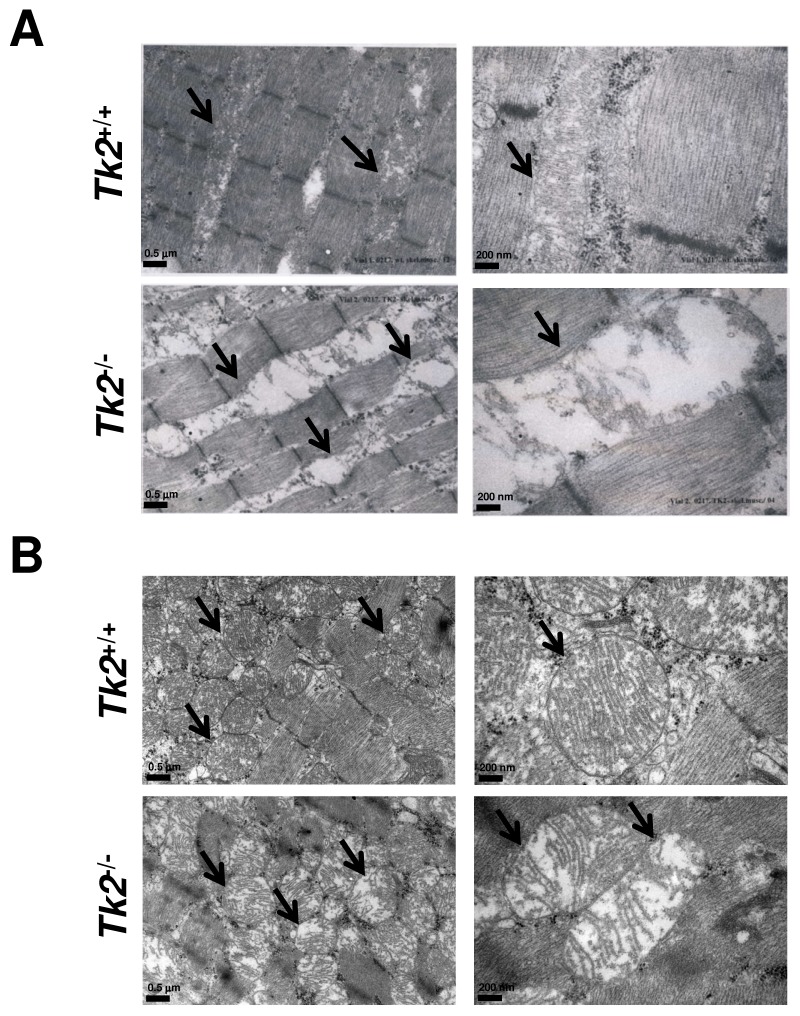
*Tk2* ^+/+^ and *Tk2* ^−/−^ mice skeletal muscle and heart mitochondria ultrastructure. Transmission electron microscopy images of skeletal muscle (**A**) and heart (**B**) sections isolated from wild-type (*Tk2* ^+/+^) and *Tk2* knockout (*Tk2* ^−/−^) 14 days-old mice. Mitochondria are indicated with solid arrows in the pictures.

We further analysed the mitochondrial phenotype both in heart and skeletal muscle of wild-type (*Tk2* ^+/+^) and *Tk2* knockout (*Tk2* ^−/−^) mice. In a previous study from our group [12], it was described that 14 days-old knockout mice did not show major skeletal muscle histological changes (Figure S1B) and had mitochondria with disrupted *cristae* structure in heart tissue. We analysed the ultrastructure of skeletal muscle and heart muscle mitochondria with transmission electron microscopy and confirmed the previous observation in heart and an even more pronounced alteration in skeletal muscle (Figure 1A and B). Mitochondria from *Tk2* ^−/−^ skeletal muscle were bigger and less dense than *Tk2* ^+/+^ mitochondria and their inside *cristae* structure was disrupted. However, mitochondria membranes were not degraded. In order to elucidate if the quantity of mitochondria was altered in these tissues, we compared by Western-blot the expression of a membrane mitochondrial protein (VDAC – voltage-dependent anion channel) and a cytoplasmic protein (β-actin). The ratio between the expression of these proteins in skeletal muscle did not differ between *Tk2* ^+/+^ and *Tk2* ^−/−^ tissues, indicating a similar quantity of mitochondria (Figure S2). However, in heart, the expression of VDAC protein seemed to be slightly increased in *Tk2* ^−/−^ tissue comparing to *Tk2* ^+/+^ heart. Another observed difference in *Tk2* knockout skeletal muscle samples was a high accumulation of lipid globules/droplets (Figure S3). These structures were not found in the *Tk2* ^+/+^ skeletal muscle samples analysed.

### Global gene expression analysis in TK2 deficient skeletal muscle and heart tissues

**Figure 2:**
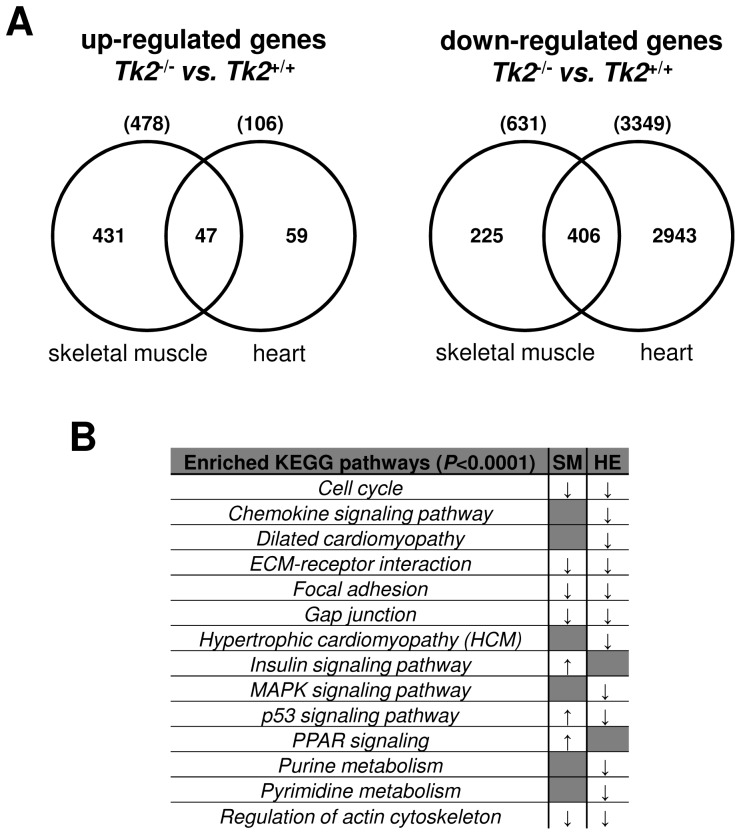
Gene expression deregulation in 11 days-old *Tk2* knockout (*Tk2* ^−/−^) skeletal muscle and heart comparing to wild-type (*Tk2* ^+/+^) tissues from mice with the same age. **A**) Considering only genes with at least 2.0-fold variation in expression and with a statistically significant *P*-value (*P*-value<0.05), the number of up- and down-regulated genes in *Tk2* ^−/−^ skeletal muscle and heart, comparing to the same tissues in *Tk2* ^+/+^ mice, are indicated. The numbers of similar genes that were up- or down-regulated in both analyses are indicated in the intersection of the Venn diagrams. **B**) In the group of differentially expressed genes in skeletal muscle and heart, statistically significant enrichment of genes belonging to specific KEGG pathways was detected using the Pathway analysis from the Expander software (see methods). Pathways significantly enriched in the group of up-regulated genes are marked with an upwards arrow (↑) and those enriched in the group of down-regulated genes are marked with a downwards arrow (↓). SM and HE indicate each of the gene expression analyses done, in skeletal muscle and in heart, respectively.

In order to understand gene expression deregulation caused by TK2 deficiency and consequent mtDNA depletion in both skeletal muscle and heart tissues, we performed an Affymetrix microarray analysis, using the Affymetrix Array plate platform Mouse Gene 1.1 ST Array. We compared the global gene expression of skeletal muscle and heart muscle harvested from 11 days old *Tk2* knockout mice (*Tk2* ^−/−^) with the global gene expression of the same tissues harvested from wild-type mice (*Tk2* ^+/+^) at the same age. Although the previous phenotypes have been observed at 14 days-old mice, we chose the age of 11 days for microarray analysis, in order to ensure that the gene expression analysis was performed in mice that were not in a bad condition, too close to the end of their lifespan. We observed that gene expression deregulation in response to TK2 deficiency was globally different in skeletal muscle and heart muscle tissues. Considering only genes with at least 2.0-fold variation in expression and with a statistically significant *P*-value (*P*-value <0.05), we detected 478 up-regulated genes and 631 down-regulated genes in skeletal muscle; for heart, 106 up-regulated genes and 3349 down-regulated genes were detected (Figure 2A; Table S1). Skeletal muscle and heart shared only 47 up-regulated genes with no statistically significant KEGG pathway enrichment (see methods). On the other hand, they shared 406 down-regulated genes, with statistically significant enrichment of genes belonging to KEGG pathways like cell cycle, ECM-receptor interaction, Focal adhesion, Gap junction and Regulation of actin cytoskeleton (Figure 2A and B; Table S1). In heart, other pathways were enriched in the group of down-regulated genes including those belonging to Chemokine signaling pathway, MAPK signaling pathway, purine and pyrimidine metabolism, and also genes involved in Dilated or Hypertrophic Cardiomyopathy (Figure 2B; Table S1).

Among the 478 up-regulated genes in skeletal muscle of *Tk2* knockout mice, we detected an enrichment of genes involved in PPAR signaling pathway, insulin signaling pathway and p53 signaling pathway (Figure 2B; Table S1). The PPARs are ligand-activated transcription factors that bind to specific DNA elements called peroxisome proliferator response elements (PPREs) in the regulatory region of a variety of genes encoding proteins that are involved in lipid metabolism and energy balance or homeostasis. Consequently, we also observed an enrichment of genes involved in fatty-acid oxidation and transport. Moreover, when we looked to the expression of PPARs target genes, several of them were increased in their expression, mainly those involved in fatty acid oxidation (*Ehhadh*, *Acaa1b*, *Scp2*, *Acox1*, *Cpt1b*, *Cpt2*, *Acadl*, *Acadm*) and transport (*Lpl*, *Acsl1*, *Slc27a1*, *Cd36*), cholesterol metabolism (*Nr1h3*, *Cyp27a1*), adipocyte differentiation (*Angptl4*, *Fabp4*, *Sorbs1*, *Adipoq*), ketogenesis (*Hmgcs2*) and ubiquitination (*Ubc*) (Table S1). Expression of almost all enzymes involved in β-oxidation was increased in skeletal muscle, which means that the breakdown process of fatty-acids to generate acetyl-coA was active in this tissue. Also several up-regulated genes in *Tk2* knockout skeletal muscle are involved in insulin signaling pathway (*Insr*, *Irs2*, *Pik3r1*, *Pdpk1*, *Akt2*, *Akt3*, *Foxo1*, *Srebf1*, *Mtor*, *Acaca*, *Acacb*, *Fasn*, *Pck2*) (Table S1). This pathway is central to regulate carbohydrate and fat metabolism in the body. With its activation follows that liver, muscle and fat cells increase their uptake of glucose from blood and decrease the use of fat as energy source.

**Figure 3:**
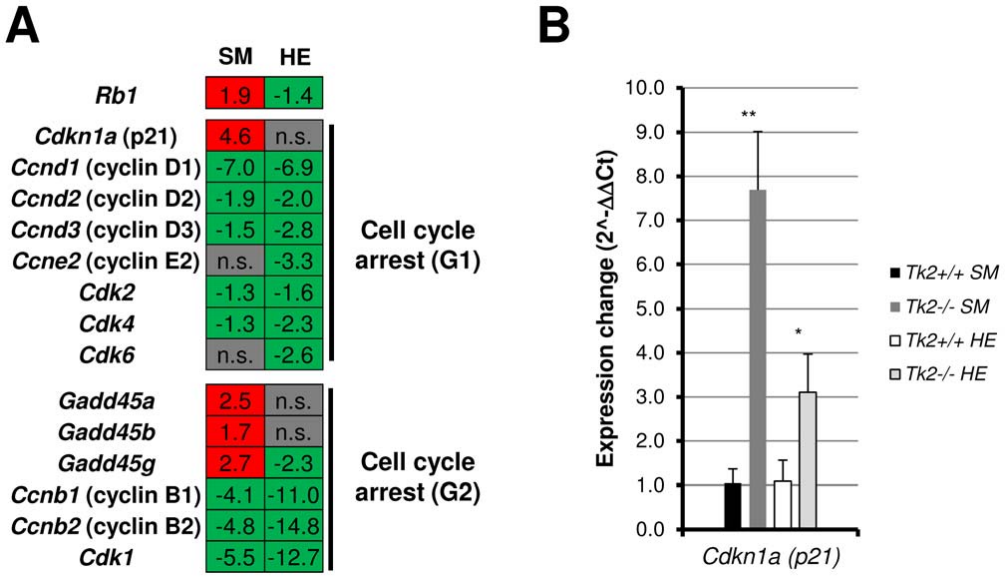
Gene expression variation of some genes involved in cell cycle arrest in both skeletal muscle and heart of 11 days-old *Tk2* knockout (*Tk2* ^−/−^) mice. **A**) Expression values obtained in the microarray analysis for some genes involved in cell cycle arrest at G1 or G2. SM and HE refer to each of the gene expression analyses made, in skeletal muscle and in heart. *n.s.* indicates that the gene is not significantly up- or down-regulated in the analysis. **B**) Expression of *Cdkn1a* (p21) was also analysed by Real-time qPCR for both tissues. The *P*-values for statistical comparisons (two-tailed unpaired Student's t-test) between *Tk2* ^+/+^ and *Tk2* ^−/−^ tissues are shown - *P<0.05; **P<0.01.

Another group of enriched genes among the 478 up-regulated genes in skeletal muscle of *Tk2* knockout mice were part of p53 signaling pathway and involved in the process of cell cycle arrest (Figures 2B; Table S1). Genes expressing p21 protein (*Cdkn1a*) and growth arrest and DNA damage inducible proteins 45 (*Gadd45a*, *Gadd45b*, *Gadd45g*) lead to cell cycle arrest at G1 or G2 when activated, by blocking the action of cyclins B, D and E. The expression of cyclins and cyclin-dependent kinase genes (*CDK*s) was decreased in skeletal muscle and also in heart (Figure 3A). Although p53 signaling pathway seemed to be activated, the gene expressing p53 protein itself (*Trp53*) was down-regulated in skeletal muscle, as well as in heart (Table S1). However, other p53 regulated target genes were up-regulated in skeletal muscle like *Fas*, *Bbc3*/PUMA, *Perp* and *Siah1a* involved in apoptosis, Sestrins (*Sesn1*, *Sesn2*) and *Dbd2*/p48 involved in DNA repair and damage prevention, and *Pten*, *Igfbp3*, *Ddit4*/REDD1, *Tsc1* and *Tsc2* involved in inhibition of Igf1/mTOR pathway (Table S1). Genes that negatively feedback the expression of p53 (*Mdm2*, *Ccng2*, *Ppm1d*/Wip1), were also up-regulated which may explain why p53 expression was decreased (Table S1). To confirm the microarray data, we analysed the expression of selected genes also by Real-time qPCR. We found that, as for other p53 target genes, *Cdkn1a*/p21 gene was significantly up-regulated in heart tissue, but in a lower range than observed in skeletal muscle (Figure 3B).

### Cell cycle and cell proliferation related genes expression is decreased in Tk2 knockout mice skeletal muscle and heart

**Figure 4:**
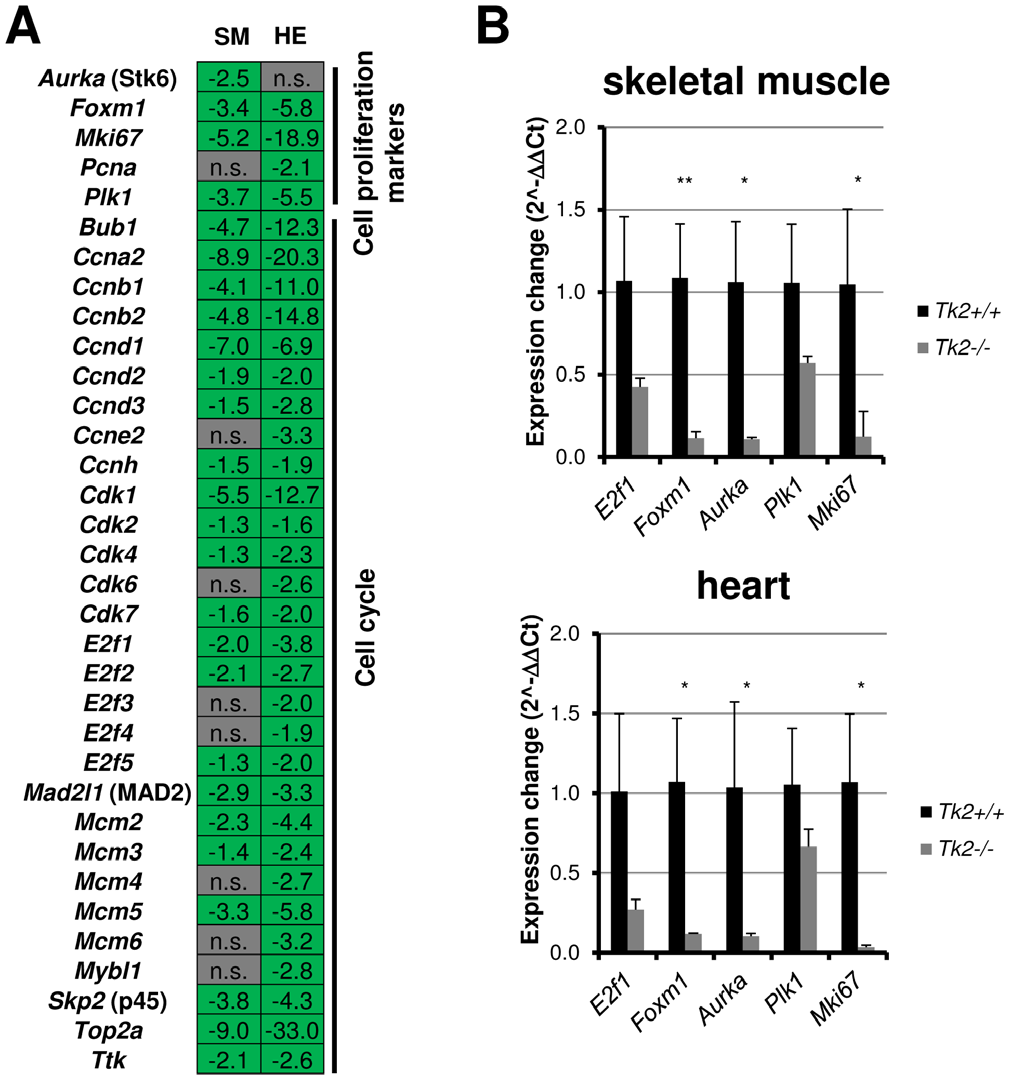
Cell cycle and cell proliferation related genes expression variation in both skeletal muscle and heart of 11 days-old *Tk2* knockout (*Tk2* ^−/−^) mice. **A**) Gene variation obtained by microarray analysis. SM and HE refer to each of the gene expression analyses made, in skeletal muscle and in heart. *n.s*. indicates that the gene is not significantly up- or down-regulated in the analysis. **B**) Expression of some genes was also analysed by Real-time qPCR. The *P*-values for statistical comparisons (two-tailed unpaired Student's t-test) between *Tk2* ^+/+^ and *Tk2* ^−/−^ tissues are shown - *P<0.05; **P<0.01.

As mentioned earlier, cell cycle and cell proliferation related genes were significantly enriched among the down-regulated genes of both skeletal muscle and heart tissues in *Tk2* knockout (*Tk2* ^−/−^) mice (Figure 2B, 3 and 4; Table S1). Cell cycle cyclins (Cyclins B and D and E) and their dependent kinases (Cdk2, Cdk4, Cdk6) were down-regulated in both skeletal muscle and heart muscle, as a result of *Cdkn1a*/p21 and *Gadd45* gene activation (Figure 3A and B). The *Rb1* gene, which expresses the retinoblastoma protein and also mediates down-activation of CDK enzymes, was up-regulated in *Tk2* ^−/−^ skeletal muscle, but not in heart (Figure 3A). Other common cell proliferation markers, usually known as proliferation signature genes, including genes involved in the fundamental process of cell proliferation, were analysed and almost all of them were highly down-regulated in both skeletal muscle and heart tissues. Some of these genes were aurora kinase A gene (*Aurka*/Stk6), E2F transcription factors genes (*E2f1* to *E2f5*), forkhead box M1 gene (*Foxm1*), *Mcm* genes (*Mcm2* to *Mcm5*), *Mki67* gene, *Pcna* gene and *Plk1* gene (Figure 4A; Table S1). To further confirm the decreased expression of these genes, we analysed selected genes by Real-time qPCR and observed a significant down-regulation in both skeletal muscle and heart of *Tk2* knockout mice (Figure 4B).

### Other dNTP pool contributing genes do not compensate for TK2 deficiency and confirm reduced proliferation in skeletal muscle and heart tissue

**Figure 5:**
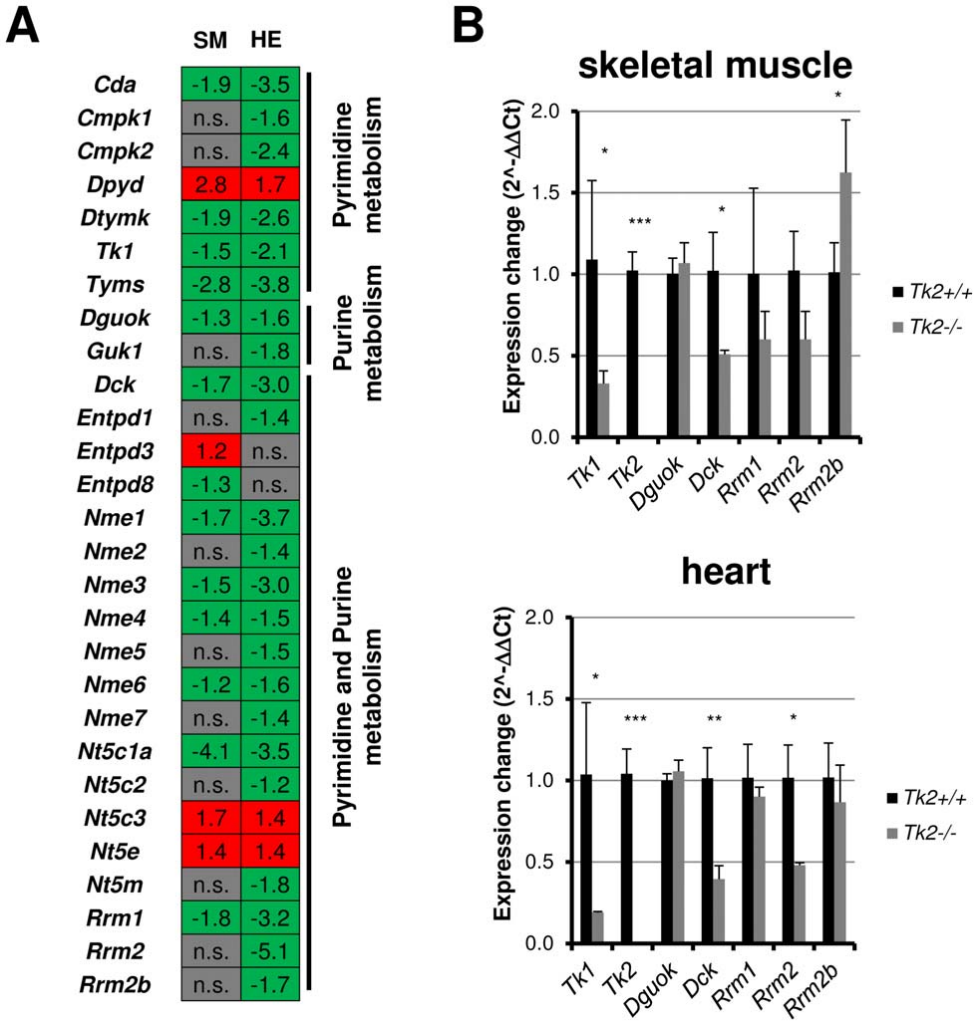
dNTP pool maintenance related genes expression variation in both skeletal muscle and heart of 11 days-old *Tk2* knockout (*Tk2* ^−/−^) mice. **A**) Gene variation obtained by microarray analysis. SM and HE refer to each of the gene expression analyses made, in skeletal muscle and in heart. *n.s.* indicates that the gene is not significantly up- or down-regulated in the analysis. **B**) Expression of deoxyribonucleoside kinases genes was also analysed by Real-time qPCR. The *P*-values for statistical comparisons (two-tailed unpaired Student's t-test) between *Tk2* ^+/+^ and *Tk2* ^−/−^ tissues are shown - *P<0.05; **P<0.01; ***P<0.001.

Since tissues lacking TK2 (*Tk2* ^−/−^) showed signs of lower proliferation rate, as compared to wild-type (*Tk2* ^+/+^) mice tissue, we decided to investigate the gene expression of other deoxyribonucleosides kinases and dNTP pool regulation enzymes, to elucidate if any compensatory dNTP synthesizing enzymes were up-regulated. An interesting observation was that in *Tk2* ^−/−^ tissues, cell cycle regulated nucleoside kinase genes were down-regulated compared to *Tk*2^+/+^ tissues (Figure 5A). It is known that expression of TK1 and dCK, as well as RNR genes (*Rmr1* and *Rmr2*), is cell cycle dependent and their expression decrease along postnatal development. However, in both *Tk2* ^−/−^ skeletal muscle and heart muscle tissues, these genes were down-regulated as early as at 11 days of age. The down-regulation of these genes was further confirmed by Real-time qPCR (Figure 5B). In addition, we also detected by Real-time qPCR that RNR-p53 subunit gene *Rmr2b* expression was significantly increased in *Tk2* ^−/−^ skeletal muscle; *Rrm2b* is not cell cycle-regulated and can contribute with some deoxynucleotides to quiescent cells, which further confirms that the population of cycling cells in TK2 deficient skeletal muscle was decreased comparing to *Tk2* ^+/+^ tissue.

### Cultured Tk2 knockout primary myoblasts proliferate slower and differentiate less than Tk2^+/+^ myoblasts, even in absence of mtDNA depletion

**Figure 6:**
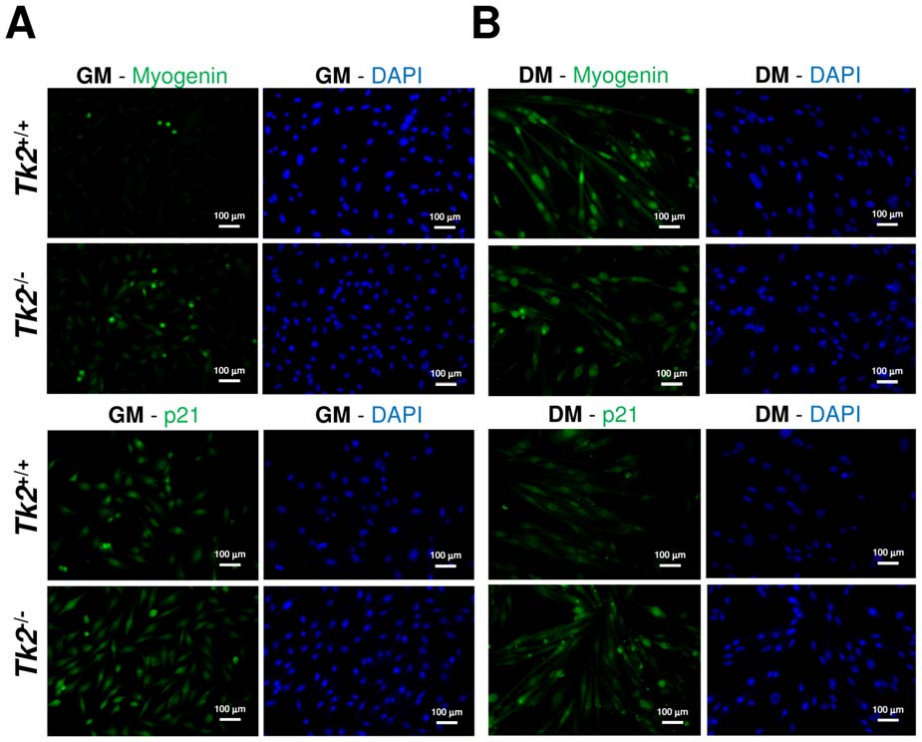
*Tk2* ^+/+^ and *Tk2* ^−/−^ primary myoblasts culture and differentiation. **A**) Primary myoblasts were isolated from 1–2 days old wild-type (*Tk2* ^+/+^) and *Tk2* knockout (*Tk2* ^−/−^) pups and cultured according to standard methods. Cultured primary myoblasts in F-10/DMEM-based primary myoblast growth medium (GM, 20% fetal bovine serum) were analysed by immunocytochemistry using anti-myogenin and anti-p21 antibodies (Abcam). Nuclei have been stained with DAPI (Sigma). **B**) Isolated primary myoblasts capacity to differentiate in myotubes was tested by changing them to differentiation medium (DM - DMEM with 5% horse serum). Immunocytochemistry was performed using anti-myogenin and anti-p21 antibodies (Abcam). Nuclei have been stained with DAPI (Sigma). Pictures were taken 2 days after myoblasts have been transferred to DM.

In order to further analyse *Tk2* knockout (*Tk2* ^−/−^) skeletal muscle, we isolated and cultured primary myoblasts from 2 days-old *Tk2* ^−/−^ pups and compared them with those from *Tk2* ^+/+^ pups. At a first sight, *Tk2* ^−/−^ myoblasts did not show any abnormal phenotype because immunocytochemistry for specific cell markers of activated and proliferating progenitor muscle cells (Desmin and Pax7) did not reveal any specific difference (Figure S4); however, when markers of myogenic differentiation (myogenin and p21) were analysed, also by immunocytochemistry, differences were detected when primary myoblasts were transferred to differentiation medium conditions (Figure 6A and B; Table 3). The increase in number of myogenin-expressing cells after change to differentiation conditions was much higher in *Tk2* ^+/+^ than in *Tk2* ^−/−^ cells; however, during growth conditions, the number of myogenin-positive muscle-compromised cells was higher in *Tk2* ^−/−^ cultures (Table 3). The percentage of p21-positive *Tk2* ^+/+^ and *Tk2* ^−/−^ cells in growth conditions was similar, but during differentiation conditions, the number of *Tk2* ^−/−^ p21-positive nuclei was increased (Table 3), indicating that a higher number of cells went into cell cycle arrest, but not necessarily differentiated into myocytes and myotubes, since a less number of cells was expressing myogenin. Also, analyzing the fusion index of these cells, we observed that *Tk2* ^−/−^ myoblasts have a lower capacity to form multinucleated myotubes than *Tk2* ^+/+^ myoblasts (Table 3), confirming their higher difficulty to differentiate.

**Table 3:** Percentage of *Tk2^+/+^* and *Tk2^−/−^* positive cells for myogenin and p21 and of nuclei contained within multinucleated myotubes (fusion index) in growth and differentiation conditions.

	Growth medium		Differentiation medium (1 day)		Differentiation medium (2 days)	
	*Tk2^+/+^*	*Tk2^−/−^*	*Tk2^+/+^*	*Tk2^−/−^*	*Tk2^+/+^*	*Tk2^−/−^*
**Myogenin**	3.3%	13.8%	42.1%	23.5%	41.5%	20.6%
**p21**	16.6%	16.4%	21.6%	31.7%	19.7%	32.7%
**Fusion index**	0.0%	0.0%	31.4%	15.0%	33.2%	12.8%

The frequency of each marker was calculated from double immunofluorescence experiments of *Tk2^+/+^* and *Tk2^−/−^* cells maintained in growth medium or exposed to differentiation medium for 1 or 2 days. The percentage of myogenin or p21 positives and the fusion index were expressed relative to the total number of DAPI stained nuclei in 10 different microscope fields (about 700–800 nuclei counted for each sample).

**Figure 7:**
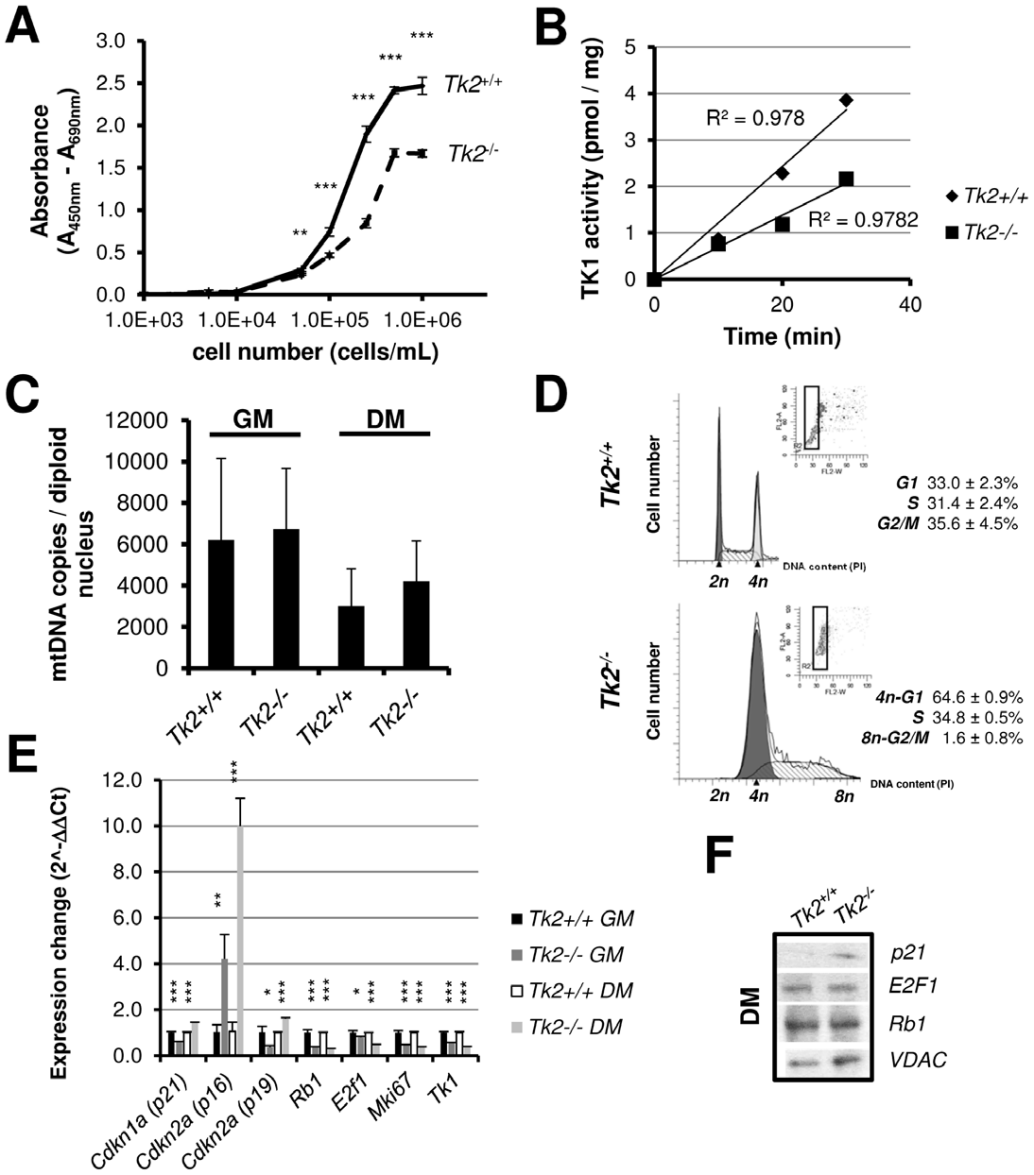
*Tk2* ^+/+^ and *Tk2* ^−/−^ primary myoblasts characterization. **A**) Growth of *Tk2* ^+/+^ and *Tk2* ^−/−^ myoblasts in F-10/DMEM-based primary myoblast growth medium was determined by XTT proliferation assay (see methods). Data points represent mean ± SD of measurements obtained from 4 independent myoblast cultures (*n* = 4). The *P*-values for statistical comparisons (two-tailed unpaired Student's t-test) between *Tk2* ^+/+^ and *Tk2* ^−/−^ data points are shown - **P<0.01; ***P<0.001. **B**) Thymidine kinase activity measured in both *Tk2* ^+/+^ and *Tk2* ^−/−^ cultured myoblasts (*n* = 2). **C**) mtDNA copy number of *Tk2* ^+/+^ and *Tk2* ^−/−^ cultured myoblasts was measured by Real-time PCR in growth (GM) and differentiation conditions (DM, 8 days). **D**) Flow cytometry analysis of *Tk2* ^+/+^ and *Tk2* ^−/−^ myoblasts cell cycle, 24 hours after cells have been plated. Data were obtained from 3 independent experiments (*n* = 3) and were analysed using the ModFit *LT* software, in order to obtain the percentages of cells in G1, S and G2/M cell cycle phases. **E**) Expression of several genes in *Tk2* ^+/+^ and *Tk2* ^−/−^ growing myoblasts (GM) and differentiated myotubes (DM, 6 days) analysed by Real-time qPCR. The *P*-values for statistical comparisons (two-tailed unpaired Student's t-test) between *Tk2* ^+/+^ and *Tk2* ^−/−^ cells are shown - *P<0.05; **P<0.01; ***P<0.001. **F**) Western-blot analysis of the expression of p21, E2F1, Rb1 and VDAC proteins in *Tk2* ^+/+^ and *Tk2* ^−/−^ differentiated myotubes (DM, 6days).

To better characterize the myoblasts phenotype, we analysed their proliferation with a XTT cell proliferation assay and detected a significant decrease in growth/proliferation of *Tk2* knockout myoblasts (Figure 7A), indicating that these cells are less metabolic active than *Tk2* ^+/+^ cells in growth conditions. We also measured TK1 activity in the isolated primary cells and observed a decreased activity in *Tk2* ^−/−^ myoblasts (Figure 7B), in accordance to what we observed before for skeletal muscle tissue. No mtDNA depletion was detected in *Tk2* knockout cells when compared with wild-type (*Tk2* ^+/+^), in both growth and differentiation conditions (Figure 7C), and cell cycle analysis using flow cytometry revealed that a big part of *Tk2* ^−/−^ myoblasts in culture are being arrested in a tetraploid state (*4n*-G1) from where some cells can escape, synthesize DNA and completely divide (Figure 7D; Figure S5), allowing the culture to grow but in a much slower rate. Gene expression analysis by Real-time qPCR of genes involved in cells cycle regulation showed an extremely high up-regulation in *Tk2* ^−/−^ cells of p16 gene (*Cdkn2a*) in both, growth and differentiation conditions (Figure 7E); it also detetcted up-regulation of p19 (*Cdkn2a*) and p21 (*Cdkn1a*) genes, in differentiation conditions. Other genes involved in cell proliferation (*Rb1*, *E2f1* and *Mki67*) and genes of deoxyribonucleosides kinases (*Tk1*) were all down-regulated in both conditions (Figure 7E). The increase in the expression of p21 protein during differentiation conditions in *Tk2* ^−/−^ cells was confirmed by Western-blot, but the decrease in E2F1 and Rb1 expression was not detectable (Figure 7F).

## Discussion

In this study we analysed the effect of TK2 deficiency in two different muscle tissues of *Tk2* knockout (*Tk2* ^−/−^) mice during their postnatal developmental phase. *Tk2* ^−/−^ pups showed pronounced growth retardation, clearly visible after 8 days of life and reaching a level of about 50% of body weight loss at 14 days of age. At this stage, skeletal muscle fibers from hind limbs were significantly underdeveloped compared to wild-type (*Tk2* ^+/+^) mice, but for heart, there was no significant differences between *Tk2* ^−/−^ and *Tk2* ^+/+^ mice. The weight loss and growth retardation was accompanied by a progressive loss of mtDNA copy number and damage of mitochondrial structural in both muscular tissues. At 14 days-old *Tk2* ^−/−^ muscle tissues had just about 30% of the mtDNA copies existing in *Tk2* ^+/+^ tissues. We also found a mild mtDNA decrease during muscle postnatal development in normal *Tk2* ^+/+^ tissues; previous studies in mouse [17] claim that mtDNA content in heart and skeletal muscle increase gradually with age; however, those analysis only started at 14 days of age and went until 15 months-old mice. No analysis before 14 days was done. In humans, several studies have already been done measuring mtDNA copy number during lifespan, but the ones that have been performed in muscle and include data during postnatal development pointed to different conclusions: those that found lack of change of mtDNA content with age [18], [19] and those that described an increase in mtDNA number with age [20], [21]. Although tremendous progress has been made in many aspects of mitochondrial biology, the regulation and variation of the mitochondria genome copy number in tissues, especially during postnatal development, is something that still needs to be further clarified.

It is known, however, that postnatal growth in mouse is rapid and highly energy demanding; usually the first 3 weeks of postnatal life is a period of intense growth, with body weight increasing 7–8 fold, half of which is accounted for by the increase in skeletal muscle [22]. Postnatal muscle growth is achieved by an increase in number (hyperplasia) and size (hypertrophy) of myofibres in mice. The new myonuclei required during postnatal muscle growth are provided by proliferation of muscle progenitor and satellite cells [23], [24], as shown by the rate of DNA-synthesis being greatest before 21 days of age, after which it declines sharply [25], [26]. In heart the picture is different in that cardiomyocytes proliferate rapidly during fetal life but exit cell cycle soon after birth, after which the predominant form of growth shifts from hyperplasia to hypertrophy [27], [28]. Previous studies suggested that most cardiac cells in rat and mouse gradually cease to undergo DNA replication, and thus proliferation, within the first two or three weeks after birth [29]–[31] and further increase of myocardial mass is not accompanied by cardiomyocyte proliferation [32]. This difference in developmental processes can, at least partly, explain the distinct effects of TK2 deficiency between skeletal muscle and heart tissue. At birth, heart is already at the post-mitotic stage of development and it is unclear if there is any cardiomyocyte proliferation at all during postnatal development [33]. Differently from heart muscle, the developmental process of skeletal muscle is intense during postnatal phase and is thus much more susceptible to growth disturbances occurring during this phase [26].

The vast gene expression deregulation in skeletal muscle and heart in response to TK2 deficiency and consequent mtDNA depletion highlighted the pathways affected during postnatal developmental phase. The down-regulation of genes involved in cell cycle progression and also of genes involved in the control of cellular activities such as adhesion, migration, differentiation, proliferation and survival were in accordance with our observations of slow growth and slow development of *Tk2* knockout mice and suggests a rigorous economy of resources that these tissues have to follow in order to survive during extreme mtDNA depletion and mitochondrial damage. The activation of PPAR and insulin signaling pathways in skeletal muscle was indicative of the energy demand of this tissue during postnatal stage. The observed gene activation shows that maintenance of glucose and lipid homeostasis, and their usage in order to produce energy, is essential for normal development, growth and survival. In accordance with this are the lipid droplets and the up-regulation of β-oxidation genes found in skeletal muscle of *Tk2* knockout mice. The association of lipid droplets with mitochondria has been observed in skeletal muscle tissue [34] and found to become more abundant when energy requirements increase, as during physical exercise [35].

The significant up-regulation of several genes related to p53 signaling pathway in skeletal muscle suggested that a substantial number of muscle cells were entering a state of cell cycle arrest. The opposite happened in heart probably because of the different postnatal development processes in heart and skeletal muscle discussed above. It is known that p53 and p53-related genes have a role in balancing development and differentiation of several kinds of tissues in mammals, including the myogenic lineage [36]. Thus, up-regulation of p53 pathway can reveal differences between the proliferation/differentiation state of cells in *Tk2* knockout skeletal muscle and *Tk2* ^+/+^ tissue from mice with the same age. Actually for some of the p53 targets, like *Cdkn1a*/p21 gene, their expression is not always p53-dependent in muscle and other terminally differentiating cells [37]. In these cases, p21 protein functions during development as an inducible growth inhibitor and can be used as a marker for cells that permanently exit cell cycle and commit to differentiation. p21 associates with cyclin-CDK complexes and high amounts of this protein inhibit CDK activity and cause growth arrest [38]–[40]. In addition, the negative regulation of cell growth by p21 has been found to be through E2F-mediated repression of transcription [41]. The inhibition of E2F gene expression by p21, in addition to up-regulated expression of *Rb1* gene, also includes alterations of Rb/E2F pathway in the mechanism of arrest and slow cell proliferation in TK2 deficient tissues. In fact, in addition to the permanent up-regulation of p21, other CDK inhibitors and negative cell cycle regulators, such as retinoblastoma gene product Rb1, contribute as markers of muscle cell differentiation [42].

The *Tk1*, *Dck* and RNR (*Rrm1* and *Rrm2*) genes were down-regulated in *Tk2* knockout muscles. *Tk1* expression is regulated by the E2F transcription factor [43], [44] and it also interacts with the inhibitor p21 [45]. It is also well established that TK1 normally is expressed in very low amounts in differentiated muscle tissues [46]. The decreased expression of *Tk1*, the increased expression of CDK inhibitor genes like *Cdkn1a*/p21 and *Rb1* and the decreased expression of major positive cell cycle regulators (cyclins and CDKs) in *Tk2* knockout muscle tissues may indicate, mainly for skeletal muscle, differences in cell composition of differentiated and non-differentiated myogenic cells. The low amount of activated myoblasts/satellite cells in skeletal muscle of *Tk2* knockout (*Tk2* ^−/−^) mice was also corroborated by the down-regulation of several stem cell and skeletal muscle progenitor cell specific markers, like *Cd34*, *Pax7*, *Sox8*, *Vcam1*, *Caveolin-1* and *Myod* (Figure S6 and Table S1).

A striking observation was that growing primary myoblasts (activated satellite cells) isolated from *Tk2* ^−/−^ skeletal muscle had already slower proliferation *in vitro* and lower TK1 activity, even in absence of mtDNA depletion, and that in differentiation conditions, mtDNA levels were also the same in *Tk2* ^+/+^ and *Tk2* ^−/−^ myotubes. The observed accumulation of *Tk2* ^−/−^ tetraploid (*4n*-G1) cells in growth conditions cultures can explain the decrease in proliferation and the less ability to differentiate of these cells. Tetraploidy generation can constitute an adaptive response to stress [47] and has already been shown to arise in response to cell senescence, genotoxic, metabolic or oxidative stresses and in response to infection by some viruses [48]. Several genes associated with cell cycle arrest were up-regulated in *Tk2* ^−/−^ cells in both, growing and differentiation conditions, like p16 gene, or just in differentiation conditions, like p21 and p19 genes. It is well established that the expression levels of p16, p18, p19 (inhibitor of CDK4 family - INK4 proteins) and of p21, p27 and p57 (cyclin dependent kinase-interacting protein/kinase inhibitory protein family - CIP/KIP proteins) all increase with differentiation [37], [49]. However, the higher expression of p16, p19 and p21 in *Tk2* ^−/−^ primary cells can also be a sign of premature senescence. Senescence induction and maintenance is usually mediated by the Arf/p53/p21 and p16/pRb tumor suppressor pathways, depending on species and cellular context [50]. Premature senescence state and tetraploidy in *Tk2* ^−/−^ myoblasts may illustrate a way to cope with the increasing metabolic stress in these cells due to their lack of TK2 and difficulty to maintain proper dNTP pools and mtDNA levels. It will be interesting to investigate, in future studies, if premature cellular senescence in *Tk2* ^−/−^ cells is caused by dNTP pool imbalance, decrease of mtDNA turnover, mitochondrial damage, oxidative stress or other alterations. In addition, we can study if *Tk2* knockout primary cells are more prone to transform, since senescence is common at pre-malignant phases of tumor development in several mouse tumor models [50]; cycling tetraploidy is usually considered a crucial step from diploidy to cancer-related chromosomal instability and from senescence to malignancy [51].

A recent study showed that mtDNA mutagenesis affected neural and hematopoietic somatic stem cells (SSCs) function in mice [52]. In a similar way, lack of TK2 in skeletal muscle SSCs (activated primary myoblasts) reduces their proliferation and competence to differentiate into proper myotubes and myofibres during mice postnatal development. In heart, the presence of dividing cells in postnatal tissue is low and thus less important; however, in skeletal muscle, the activated satellite cells pool is of extreme importance for correct muscle development and growth [53], [54].

## Materials and Methods

### Ethics statement

Official Swedish regulations for use and care of laboratory animals were followed throughout and the experimental protocol was approved by the local ethical committee (*Jordbruksverket*, ethical permission numbers S104-09 and S135-11).

### Mice

Thymidine kinase 2 (*Tk2*) knockout mouse was already previously reported and characterized [12]. Mating heterozygous *Tk2* ^+/−^ males and females produced litters with wild-type (*Tk2* ^+/+^), heterozygous (*Tk2* ^+/−^) and homozygous knockout (*Tk2* ^−/−^) pups with expected Mendelian frequency. For these experiments, we sacrificed mice with 4, 8, 11 and 14 days-old and skeletal muscle and heart have been harvested.

### mtDNA copy number quantification

The number of mtDNA copies per diploid nucleus in mouse tissues was determined using real-time PCR absolute quantification, using an ABI 7500 Fast system (Applied Biosystems). Total genomic DNA was purified from mouse tissues using the DNeasy Blood and tissue kit (QIAGEN). About 10 ng of gDNA were used in each reaction. Primers and probe for mouse *mt-Nd1* gene (mitochondrially encoded NADH dehydrogenase 1; forward primer: 5′-TCGACCTGACAGAAGGAGAATCA, reverse primer: 5′-GGGCCGGCTGCGTATT, probe: **FAM**-AATTAGTATCAGGGTTTAACG-**TAMRA**) and for single-copy mouse *Rpph1* gene (nuclear encoded ribonuclease P RNA component H1; forward primer: 5′-GGAGAGTAGTCTGAATTGGGTTATGAG, reverse primer: 5′-CAGCAGTGCGAGTTCAATGG, probe: **FAM**-CCGGGAGGTGCCTC-**TAMRA**) were designed for this purpose. For each DNA sample, mitochondrial gene *mt-Nd1* and nuclear gene *Rpph1* were quantified separately. Standard curves have been done using known copies of a plasmid containing one copy of each of those two mouse genes referred above. According to the standard curve, the number of copies from each gene was calculated for each sample and the number of mtDNA copies per diploid nucleus was calculated according to the formula: mtDNA copies per diploid nucleus  = 2×(*mt-Nd1* gene copies/*Rpph1* gene copies).

### Transmission electron microscopy

Hind limb skeletal muscles and hearts from 14 days-old mice have been harvested and fixed immediately with freshly made 2.5% glutaraldehyde, postfixed in 1% osmium tetroxide, embedded in Epon, and post-stained with 1% uranyl acetate. Ultramicrotome sections were made approximately 60 nm thick. Ultra-samples were analysed in a Hitatchi H-7100 electron microscope at 75 kV accelerating voltage [55], [56].

### Immunoblotting analysis

Total protein from skeletal muscle and heart was extracted using RIPA buffer (50 mM Tris-HCl pH 7.6, 150 mM NaCl, 1% Nonidet P-40, 0.5% sodium deoxycholate, 0.1% SDS and protease inhibitors). Total protein concentrations were measured using the colorimetric Protein Assay (Bio-Rad). The same amount of proteins (30 µg) for each sample was run in NuPAGE 4–12% Bis-Tris gels (Invitrogen) and blotted to a Hybond-P membrane (GE Healthcare). VDAC protein and β-actin proteins have been detected using anti-VDAC (Calbiochem) and anti-β-actin (Sigma) primary antibodies. p21, E2F1 and Rb1 proteins have been detected using anti-p21 (Abcam), anti-E2F1 (Abcam) and anti-Rb1 (BD Biosciences) primary antibodies. Protein-antibody interactions were detected with a peroxidase-conjugated secondary antibody (GE Healthcare), subjected to Amersham ECL Western blotting detection reagents (GE Healthcare).

### Gene expression analysis by microarrays

Gene expression profiles from hind limb skeletal muscles and hearts from 3 different 11 days-old knockout (*Tk2* ^−/−^) mice were compared with profiles from the same tissues collected from 3 different wild-type (*Tk2* ^+/+^) mice with the same age. This was performed using Affymetrix platform and the Mouse Gene 1.1 ST Array. Total RNA was extracted with Rneasy kit (QIAGEN) and Ambion WT Expression kit (Invitrogen) have been used for labeling. Labeling, hybridization and scanning were performed according to manufacturer's instructions. Data were normalized and in order to determine those genes that were differentially expressed between *Tk2* ^−/−^ and *Tk2* ^+/+^ tissues, a t-test was applied. KEGG pathway (http://www.genome.jp/kegg/pathway.html) enrichment was performed using the Pathways Analysis tool included in Expander software [57], [58]. The microarray data presented in this publication have been deposited in NCBI's Gene Expression Omnibus [59] and are accessible through GEO Series accession number GSE38449 (http://www.ncbi.nlm.nih.gov/geo/query/acc.cgi?acc=GSE38449).

### Real time qPCR analysis

Total RNA was extracted with RNeasy kit (QIAGEN) and cDNA was synthesized using High Capacity cDNA Reverse Transcription Kit (Applied Biosystems). qPCR analysis was performed in the ABI 7500 Fast machine (Applied Biosystems) using appropriate primers for each gene. Platinum SYBR Green qPCR SuperMix-UDG (Invitrogen) was used in qPCR analysis of cell proliferation marker genes and Taqman Universal PCR Master Mix with specific Taqman probes (Eurofins-MWG) were used in qPCR analysis of deoxyribonucleoside kinases genes. The housekeeping gene *Gapdh* was used as loading control for both analyses. In order to obtain expression variation, the 2^−ΔΔCt^ method has been used.

### Mouse primary myoblast isolation and culture

Primary myoblasts from wild-type (*Tk2* ^+/+^) and knockout (*Tk2* ^−/−^) mice have been prepared and cultured as previously described [60]. Shortly, 2 days-old, *Tk2* ^+/+^ and *Tk2* ^−/−^ pups have been sacrificed and their limbs isolated. Muscles from the limbs were dissected away from the skin and bone in a drop of sterile PBS on ice. Muscles have been minced for several minutes in a collagenase/dispase/CaCl_2_ solution and then incubated at 37°C for about 20 minutes. After centrifuge cells for 5 minutes at 350 *g*, the pellets were resuspended in F-10-based primary myoblast growth medium (20% fetal bovine serum; GIBCO) and plated in previously prepared collagen coated culture dishes. Selective growth in F-10-based primary myoblast growth medium and special passaging conditions allowed the myoblasts to become the dominant cell type in the culture over the fibroblasts within 3 weeks. After the fibroblasts were no longer visible in the culture, the medium has been changed to F-10/DMEM-based primary myoblast growth medium (20% fetal bovine serum; GIBCO). Differentiation of myoblasts into myotubes was made changing cells to differentiation medium (DMEM with 5% horse serum; GIBCO). Cell proliferation in F-10/DMEM-based primary myoblast growth medium for both cell types prepared was measured using the colorimetric XTT proliferation assay (Cell proliferation kit II – XTT, Roche), according to manufacturer's instructions and using a 4 hours incubation with XTT labeling mixture. The assay is based on the cleavage of the yellow tetrazolium salt XTT to form an orange formazan dye by metabolic active cells. Basically, serial dilutions of *Tk2* ^+/+^ and *Tk2* ^−/−^ cells, grown for 36 hours after seeding in a 96 well tissue culture plate, were incubated with the yellow XTT solution (0.3 mg/mL) for 4 hours. After this incubation period, the orange formazan solution was formed and spectrophotometrically quantified using a MCC/340 ELISA plate reader (Labsystems Multiskan). An increase in number of proliferating cells results in an increase in the overall activity of mitochondrial dehydrogenases in the sample. This increase directly correlates to the amount of orange formazan formed, as monitored by the absorbance.

### Immunocytochemistry

Myoblasts or myotubes in cell culture were briefly rinsed with phosphate-buffered saline (PBS), fixed in ice-cold methanol for 10 minutes and then washed again in PBS. Then, in order to permeabilize the cells, they were incubated for 10 minutes in PBS containing 0.25% Triton X-100 and washed again. After, cells were first incubated with 1% BSA in PBST for 30 minutes to block unspecific binding of antibodies, then in the diluted primary antibody (1∶500) overnight at 4°C and, in the end, with the diluted secondary antibody (1∶500) for 1 hour at room temperature in dark. Cells were washed once more and incubated on 0.2 µg/mL of DAPI solution for 5 minutes, in order to stain DNA. The preparation was then analysed under a fluorescence microscope (Nikon Eclipse E600). Cells were analysed using anti-myogenin, anti-p21, anti-desmin and anti-Pax7 primary antibodies (Abcam) and Alexa Fluor 488 anti-rabbit or anti-mouse secondary antibodies (Molecular Probes). Nuclei have been stained with DAPI (Sigma).

### Flow cytometry

Myoblasts (about 10^6^ cells) growing in culture were suspended in PBS and then fixed in 70% ethanol for at least 2 hours at 4°C. After centrifugation, cells were washed in PBS and centrifuged again. Then the pellet was suspended in 1 mL of propidium iodide (PI) staining solution (0.1% Triton X-100, 10 µg/mL Sigma PI solution, 100 µg/mL DNase-free RNase A in PBS) and incubated at room temperature for 30 minutes in the dark. Samples were transferred to the flow cytometer (FACSCAlibur, BD Biosciences) and cell fluorescence was measured.

### Thymidine kinase activity assay

Thymidine phosphorylation assays were carried out as described before [61], [62]. Briefly, tissues were homogenized and suspended in extraction buffer [50 mM Tris-HCl pH 7.6, 2 mM dithiothreitol (DTT), 5 mM benxamidine, 0.5 mM phenylmethylsulfonyl fluoride (PMSF), 20% glycerol and 0.5% Nonidet P40]. The suspension was centrifuged at 16000 *g* for 20 minutes and the supernatant collected and stored at −80°C. The enzymatic assays were performed in 50 mM Tris-HCl (pH 7.6), 5 mM MgCl_2_, 5 mM ATP, 2 mM DTT, 15 mM NaF, 0.5 mg/ml BSA, 40–50 µg protein, 3 µM [methyl-^3^H]thymidine (Moravek) and 7 µM unlabelled thymidine in a volume of 50 µL. 10 µL of reaction mixture was spotted on Whatman DE-81 filter discs after incubation at 37°C at different time points (0, 10, 20 and 30 minutes). The filters were washed three times in 5 mM ammonium formate and the filter bound product was eluted from the filter with 0.1 M KCl and 0.1 M HCl. The radioactivity was quantified by scintillation counting using 3 mL scintillation buffer.

## Supporting Information

Figure S1 ***Tk2*** **^+/+^ and** ***Tk2*** **^−/−^ mice hind limb and heart sizes comparison and skeletal muscle histology.** **A**) Pictures of hind limbs and hearts from 14 days-old wild-type (*Tk2* ^+/+^) and *Tk2* knockout (*Tk2* ^−/−^) mice were taken immediately after they have been sacrificed. **B**) Histological analysis of skeletal muscle tissue from 14 days-old *Tk2* ^+/+^ and *Tk2* ^−/−^ mice (hematoxylin-eosin staining).
(TIF)

Figure S2 **Expression analysis of two different proteins in** ***Tk2*** **^+/+^ and** ***Tk2*** **^−/−^ skeletal muscle and heart.** Expression of a membranar mitochondrial protein (VDAC – voltage-dependent anion channel) and a cytoplasmic protein (β-actin) was analysed by western-blot. Both proteins are encoded by the nuclear genome. The ratio between the expressions of both proteins for each sample was calculated using ImageJ software.
(TIF)

Figure S3 ***Tk2*** **^−/−^ mice skeletal muscle have several lipid droplets in its ultrastructure.** Transmission electron microscopy images of skeletal muscle sections isolated from *Tk2* knockout (*Tk2* ^−/−^) 14 days-old mice. Lipid droplets are indicated with dashed arrows in the pictures.
(TIF)

Figure S4 **Desmin and Pax7 expression during** ***Tk2*** **^+/+^ and** ***Tk2*** **^−/−^ primary myoblasts growth and differentiation.** **A**) *Tk2* ^+/+^ and *Tk2* ^−/−^ primary myoblasts in F-10/DMEM-based primary myoblast growth medium (GM, 20% fetal bovine serum) were analysed by immunocytochemistry using anti-desmin and anti-Pax7 antibodies (Abcam). Nuclei have been stained with DAPI (Sigma). **B**) *Tk2* ^+/+^ and *Tk2* ^−/−^ cells after 6 days in differentiation medium (DM - DMEM with 5% horse serum). Immunocytochemistry was performed using anti-desmin and anti-Pax7 antibodies (Abcam). Nuclei have been stained with DAPI (Sigma).
(TIF)

Figure S5 **Flow cytometry analysis of** ***Tk2*** **^+/+^ and** ***Tk2*** **^−/−^ myoblasts cell cycle, 16, 24 and 36 hours after cells have been plated.** Data were obtained from 3 independent experiments (*n* = 3) and were analysed using the ModFit *LT* software, in order to obtain the percentages of cells in G1, S and G2/M cell cycle phases.
(TIF)

Figure S6 **Gene expression variation of some stem cell and muscle progenitor cell marker genes in skeletal muscle of 11 days-old** ***Tk2*** **knockout (** ***Tk2*** **^−/−^) mice.** Expression values obtained in the microarray analysis performed. SM refers to the analysis made in skeletal muscle.
(TIF)

Table S1 **Global gene expression variation in skeletal muscle (SM) and heart (HE) harvested from 11 days old** ***Tk2*** **knockout mice (TK2-) comparing to wild-type mice (WT) at the same age.** Fold-changes and *P*-values obtained in the microarray analysis performed are indicated for each gene. *ns* indicates that the gene was not significantly up- or down-regulated in the analysis.
(XLS)
