# Supplementary figures and images for: Gene Expression Deregulation in Postnatal Skeletal Muscle of TK2 Deficient Mice Reveals a Lower Pool of Proliferating Myogenic Progenitor Cells
Source: PLoS One. 2013 Jan 14;8(1):e53698. doi: 10.1371/journal.pone.0053698 (PMC3544874; doi:10.1371/journal.pone.0053698)

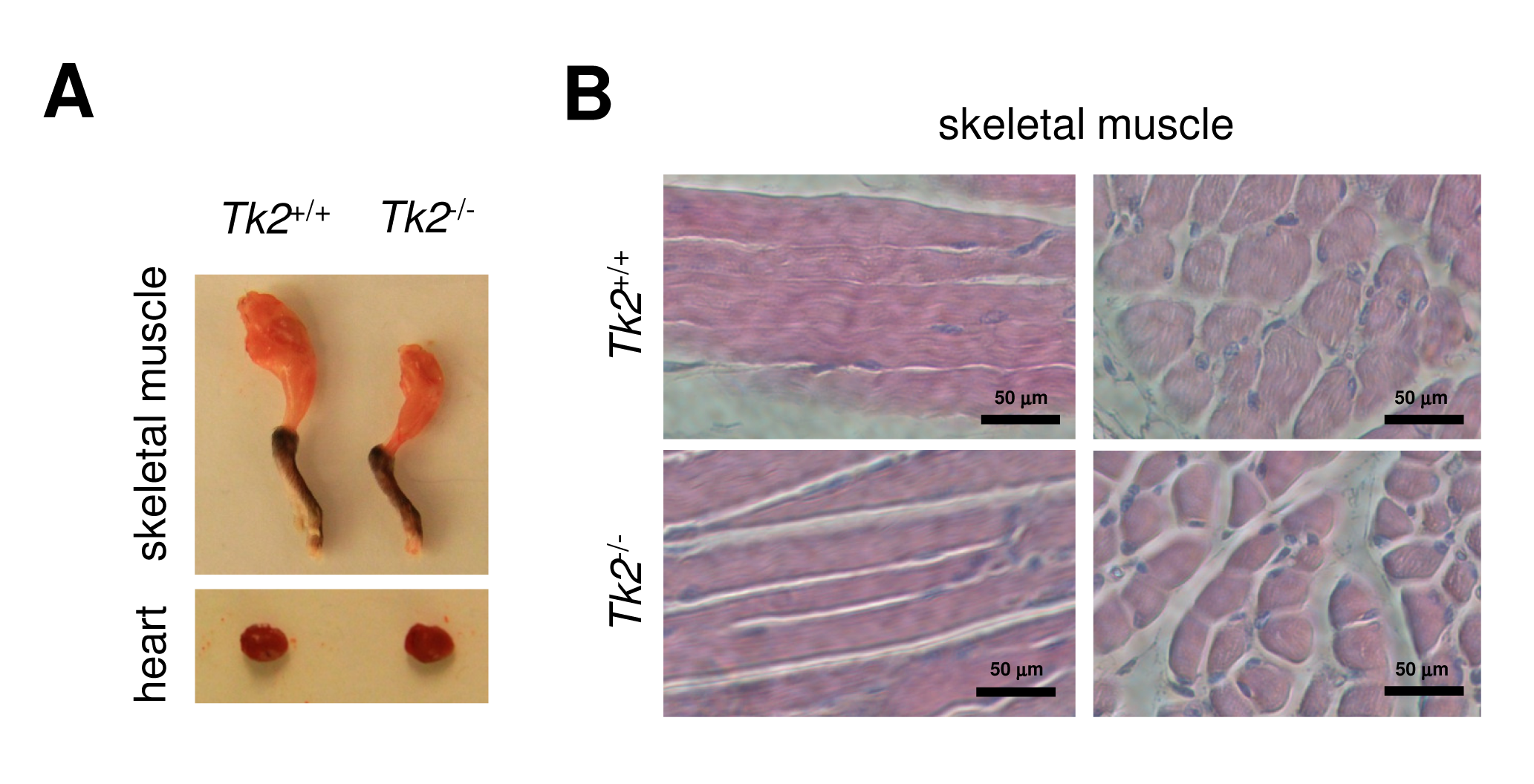

Supplement: Figure S1 — Tk2 +/+ and Tk2 −/− mice hind limb and heart sizes comparison and skeletal muscle histology. A) Pictures of hind limbs and hearts from 14 days-old wild-type (Tk2 +/+) and Tk2 knockout (Tk2 −/−) mice were taken immediately after they have been sacrificed. B) Histological analysis of skeletal muscle tissue from 14 days-old Tk2 +/+ and Tk2 −/− mice (hematoxylin-eosin staining). (TIF) [file pone.0053698.s001.tif]

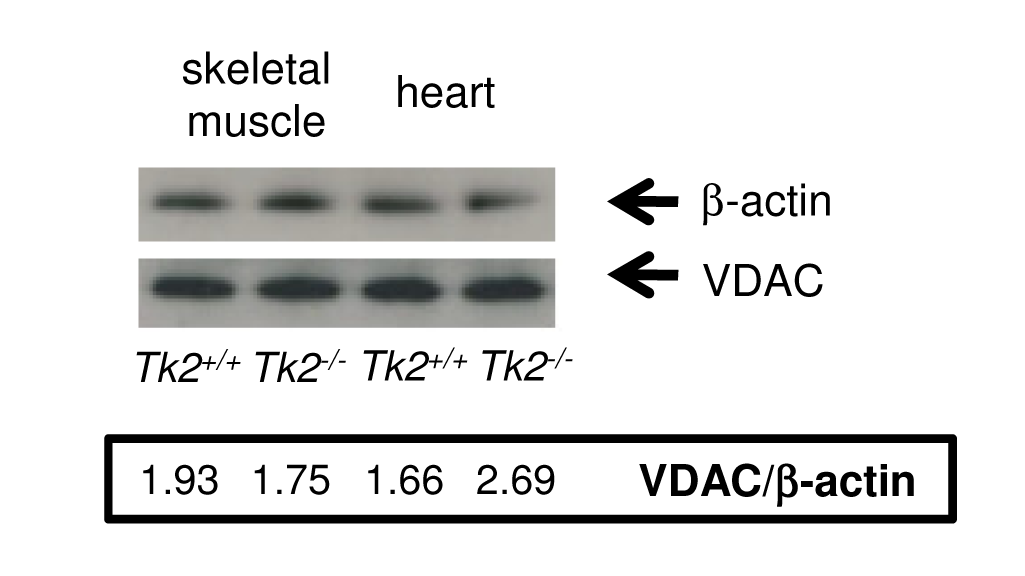

Supplement: Figure S2 — Expression analysis of two different proteins in Tk2 +/+ and Tk2 −/− skeletal muscle and heart. Expression of a membranar mitochondrial protein (VDAC – voltage-dependent anion channel) and a cytoplasmic protein (β-actin) was analysed by western-blot. Both proteins are encoded by the nuclear genome. The ratio between the expressions of both proteins for each sample was calculated using ImageJ software. (TIF) [file pone.0053698.s002.tif]

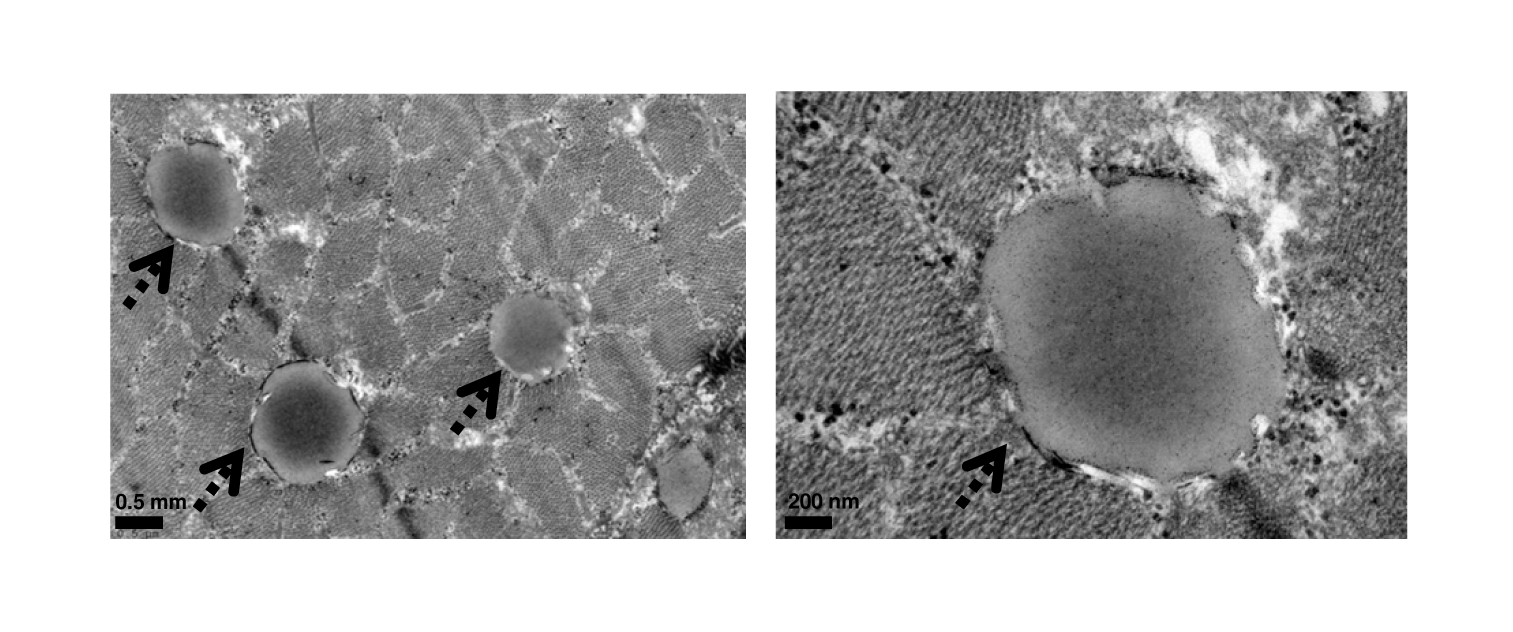

Supplement: Figure S3 — Tk2 −/− mice skeletal muscle have several lipid droplets in its ultrastructure. Transmission electron microscopy images of skeletal muscle sections isolated from Tk2 knockout (Tk2 −/−) 14 days-old mice. Lipid droplets are indicated with dashed arrows in the pictures. (TIF) [file pone.0053698.s003.tif]

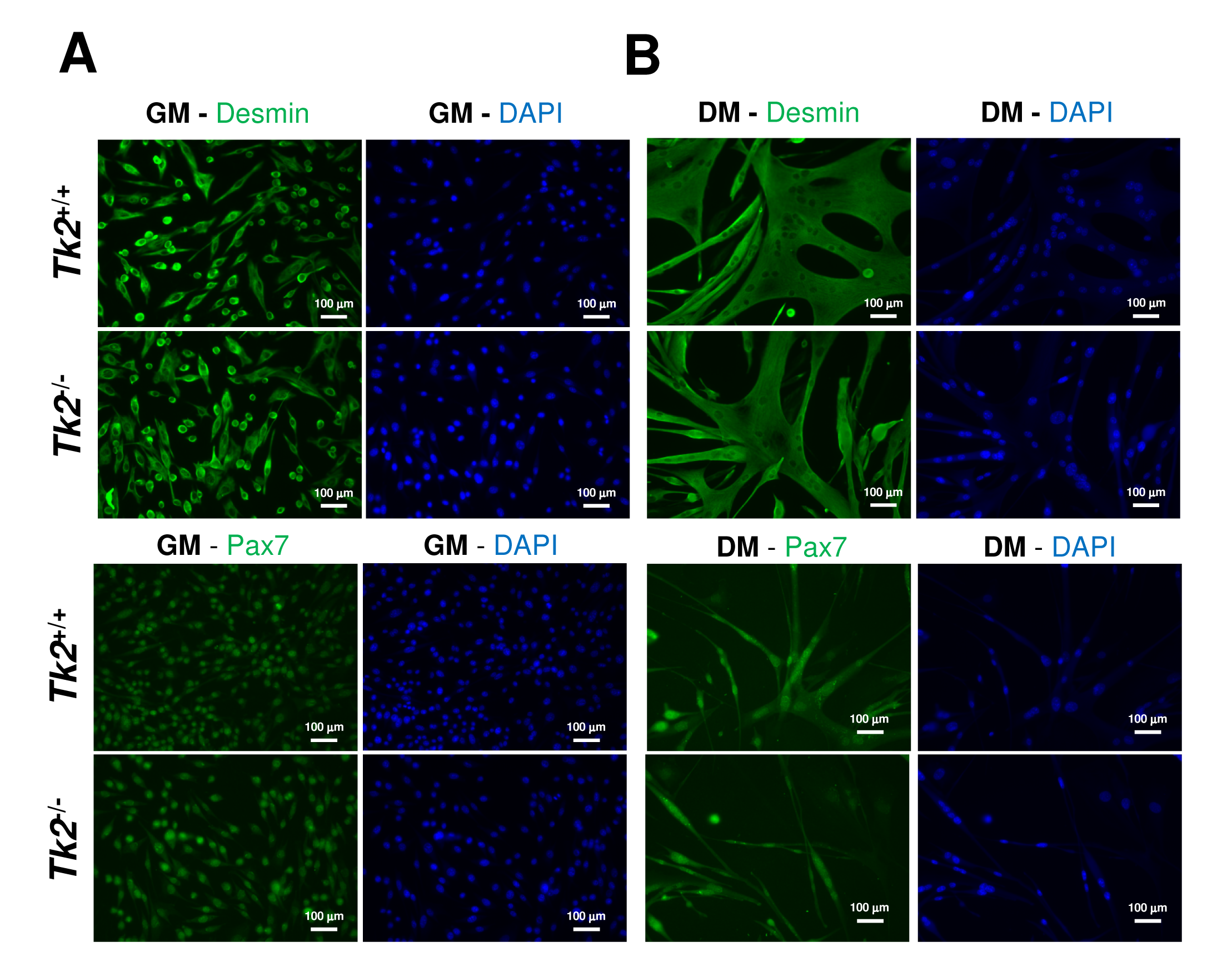

Supplement: Figure S4 — Desmin and Pax7 expression during Tk2 +/+ and Tk2 −/− primary myoblasts growth and differentiation. A) Tk2 +/+ and Tk2 −/− primary myoblasts in F-10/DMEM-based primary myoblast growth medium (GM, 20% fetal bovine serum) were analysed by immunocytochemistry using anti-desmin and anti-Pax7 antibodies (Abcam). Nuclei have been stained with DAPI (Sigma). B) Tk2 +/+ and Tk2 −/− cells after 6 days in differentiation medium (DM - DMEM with 5% horse serum). Immunocytochemistry was performed using anti-desmin and anti-Pax7 antibodies (Abcam). Nuclei have been stained with DAPI (Sigma). (TIF) [file pone.0053698.s004.tif]

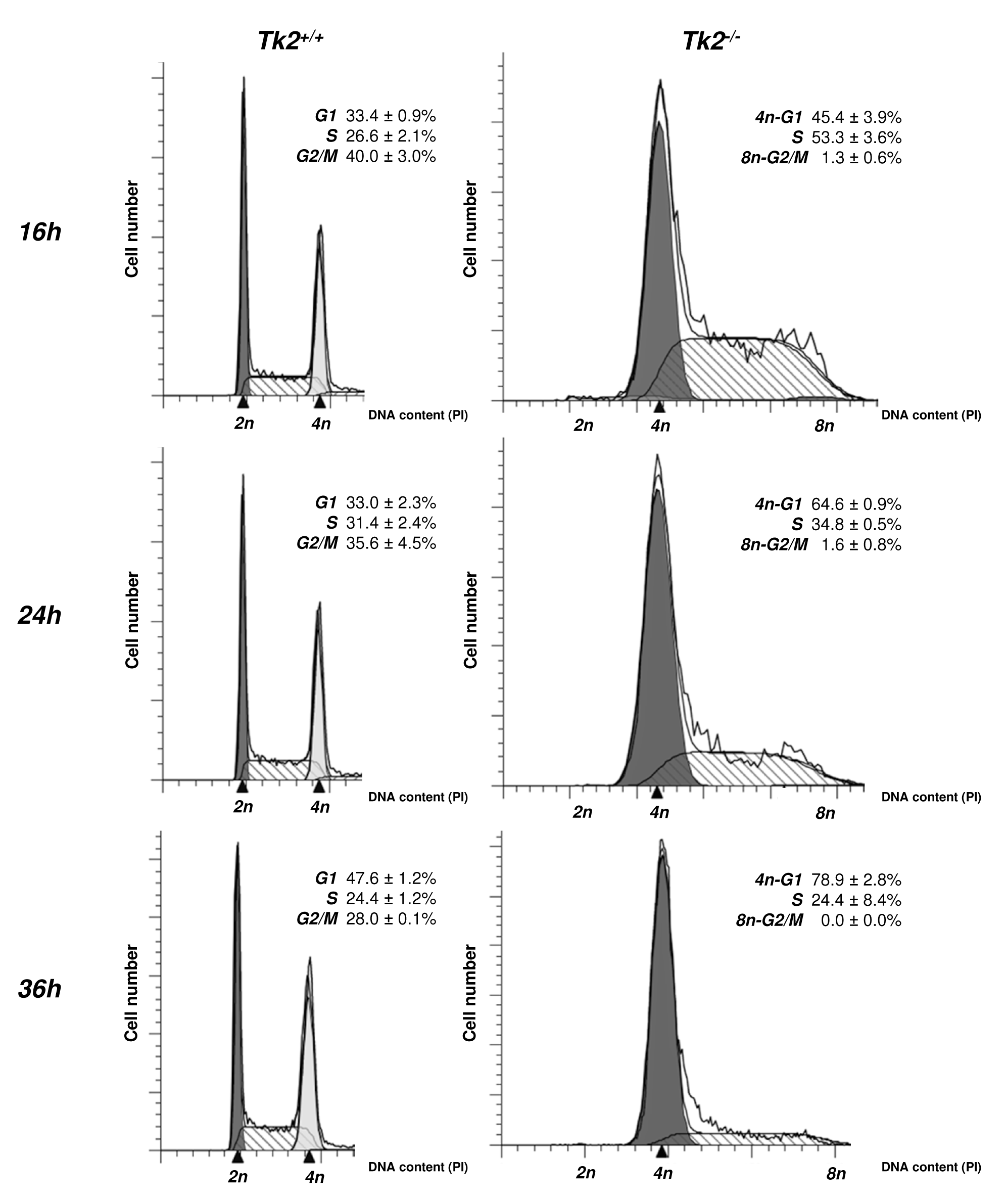

Supplement: Figure S5 — Flow cytometry analysis of Tk2 +/+ and Tk2 −/− myoblasts cell cycle, 16, 24 and 36 hours after cells have been plated. Data were obtained from 3 independent experiments (n = 3) and were analysed using the ModFit LT software, in order to obtain the percentages of cells in G1, S and G2/M cell cycle phases. (TIF) [file pone.0053698.s005.tif]

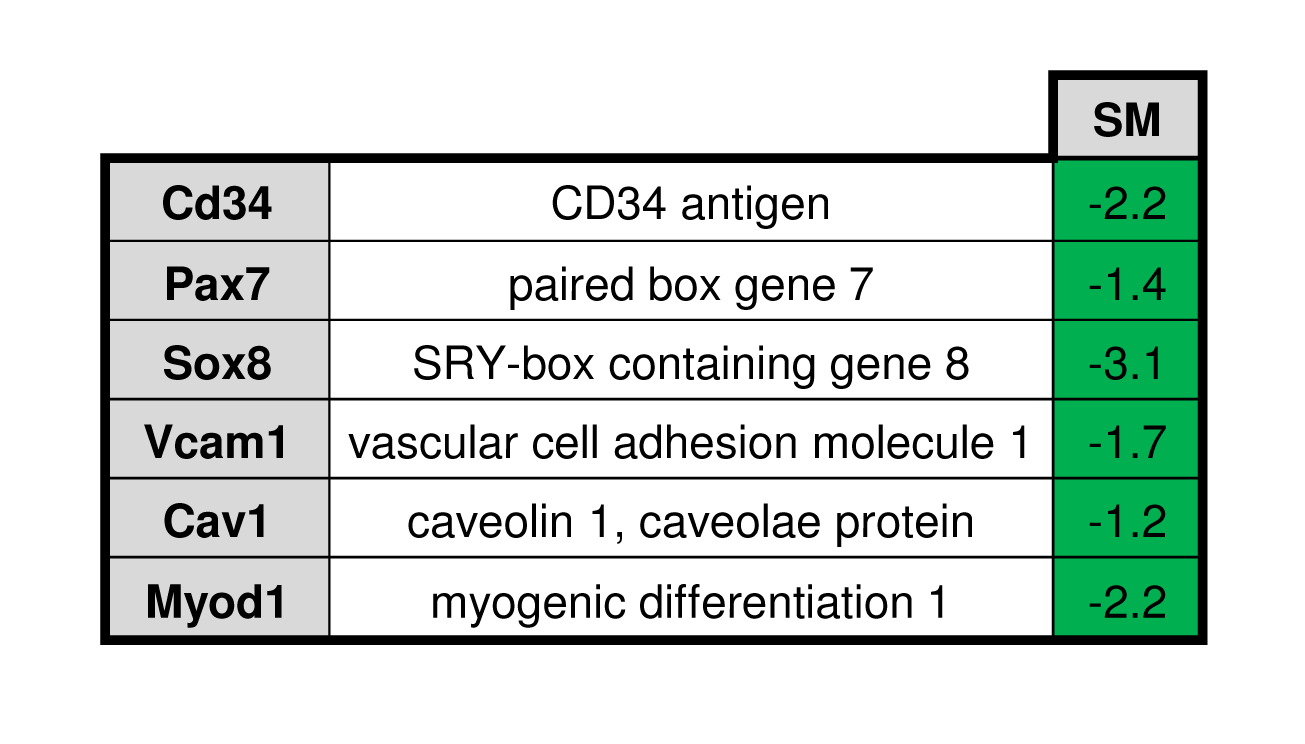

Supplement: Figure S6 — Gene expression variation of some stem cell and muscle progenitor cell marker genes in skeletal muscle of 11 days-old Tk2 knockout ( Tk2 −/−) mice. Expression values obtained in the microarray analysis performed. SM refers to the analysis made in skeletal muscle. (TIF) [file pone.0053698.s006.tif]
